# SFRP2 Is Associated with Increased Adiposity and VEGF Expression

**DOI:** 10.1371/journal.pone.0163777

**Published:** 2016-09-29

**Authors:** Rachel K. Crowley, Michael W. O’Reilly, Iwona J. Bujalska, Zaki K. Hassan-Smith, Jonathan M. Hazlehurst, Danielle R. Foucault, Paul M. Stewart, Jeremy W. Tomlinson

**Affiliations:** 1 St Vincent’s University Hospital & University College Dublin, Dublin, Ireland; 2 Institute of Metabolism and Systems Research, University of Birmingham, Birmingham B15 2TT, United Kingdom; 3 University of Leeds School of Medicine, Leeds LS2 9NL, United Kingdom; 4 Oxford Centre for Diabetes, Endocrinology and Metabolism, University of Oxford, Oxford OX3 7LJ, United Kingdom; Tohoku University, JAPAN

## Abstract

**Aims:**

The aim of this study was to assess depot-specific expression and secretion of secreted frizzled-related protein 2 (sFRP2) by adipose tissue and its effect on adipocyte biology. We measured serum sFRP2 concentrations in 106 patients *in vivo* to explore its relationship to fat mass, glycaemia and insulin resistance.

**Methods:**

Expression of sFRP2 in mouse and human tissues was assessed using polymerase chain reaction and Western blot. Western blot confirmed secretion of sFRP2 by adipose tissue into cell culture medium. Effects of recombinant sFRP2 on lipogenesis and preadipocyte proliferation were measured. Preadipocyte expression of the angiogenic genes vascular endothelial growth factor (VEGF) and nuclear factor of activated T-cells 3 (NFATC3) was measured after recombinant sFRP2 exposure. Complementary clinical studies correlating human serum sFRP2 with age, gender, adiposity and insulin secretion were also performed.

**Results:**

sFRP2 messenger RNA (mRNA) was expressed in mouse and human adipose tissue. In humans, sFRP2 mRNA expression was 4.2-fold higher in omental than subcutaneous adipose. Omental adipose tissue secreted 63% more sFRP2 protein than subcutaneous. Treatment with recombinant sFRP2 did not impact on lipogenesis or preadipocyte proliferation but was associated with increased VEGF mRNA expression. In human subjects, circulating insulin levels positively correlated with serum sFRP2, and levels were higher in patients with abnormal glucose tolerance (34.2ng/ml) compared to controls (29.5ng/ml). A positive correlation between sFRP2 and BMI was also observed.

**Conclusions:**

Circulating sFRP2 is associated with adipose tissue mass and has a potential role to drive adipose angiogenesis through enhanced VEGF expression.

## Introduction

Truncal adiposity is a risk factor for type 2 diabetes mellitus, but the nature of the relationship between adipose tissue (AT) mass and pancreatic beta cell function is not clear. The association between insulin resistance and fat mass suggests that changes in the adipocytokine milieu may have a role in the regulation of insulin secretion or a paracrine effect on adipocyte function.

The Wnt (wingless/integrated) signalling system acts by binding and activating cell surface frizzled receptors. Wnt signalling plays an important role in morphogenesis of several organ systems [1, 2] including prenatal pancreatic beta cell development [3] and postnatal beta cell function and proliferation [4–6]; and in pathological diseases such as cancer [7] and cardiac fibrosis [8]. Several studies confirmed a pivotal role of Wnt signalling in decreasing adipogenesis through down regulation of PPAR-gamma (peroxisome proliferator-activated receptor-gamma) and C/EBPs (CCAAT enhancer binding proteins) expression [9–11].

Secreted frizzled-related proteins (sFRPs) form a family of secreted glycoproteins; these are circulating soluble proteins that contain a cysteine-rich domain with homology for cell surface frizzled receptors. sFRPs are thought to act as pre-receptor Wnt antagonists by binding circulating Wnt ligands. The action of individual sFRPs has been investigated in animal models: treatment with recombinant Wnt3a protein activated Wnt signalling and increased cell expansion of mouse insulinoma cell line (MIN6) [5], rat INS1 cell line [12] and primary mouse islet cells [6], and this effect was reversed by co-treatment with sFRP1 [6]. sFRP5 is decreased in the pancreas of obese rats and humans, and its gene silencing activates the Wnt signalling pathway and promotes beta cell proliferation [13]. A recent cross-sectional clinical study of serum sFRP4 in humans with type 2 diabetes mellitus compared to pre-diabetes and normal glucose tolerance subjects revealed higher sFRP4 in subjects with diabetes and a positive correlation between sFPR4 and age, insulin levels, HbA1c and triglycerides [14]. Secreted frizzled-related protein 2 (sFRP2) belongs to this glycoprotein family, and its mRNA was shown to be present in human preadipocytes, rodent AT [15] and human AT [16]. The findings of cancer studies suggest that sFRP2 acts as a Wnt agonist in certain tumour tissues [17] and that it can be a Wnt agonist or antagonist at different tissue concentrations. In tissues with low levels of sFRP2 expression, sFRP2 appears to augment Wnt signalling and to inhibit Wnt at higher levels, as has been reported also with sFRP1 [8, 18].

Recently, mRNA expression of 5 sFRP family members was characterised in human AT where the sFRP2 mRNA levels in subcutaneous adipose tissues were positively correlated with insulin resistance [16]. These previous findings suggested that sFRP2 could have effects on adipose tissues. No human study has reported associations between circulating sFRP2 and glycaemic status nor sFRP2 in humans with a range of different body mass indices. Therefore the previous studies did not establish whether sFRP2 production by adipose tissue was of benefit in glucose homeostasis nor define the effect of sFRP2 on the adipocyte.

We have performed a series of *in vitro* experiments using animal and human cells to assess the depot-specific expression and secretion of sFRP2 by adipose tissue and to explore its potential role in adipocyte metabolism, glucose and lipid homeostasis, and insulin resistance. Importantly, we have translated our findings into a clinical setting by examining sFRP2 levels in the serum of 106 patients with a range of BMI and glucose tolerance.

## Methods

### Human Subjects

Paired fasting insulin and glucose levels were measured in 106 subjects [69 female; median BMI 28.9 kg/m^2^ (range 19.0–43.9); median age 42 years (20–67)]. Fifty-three of these subjects underwent dual energy X-ray absorptiometry (DXA) measurements of adipose tissue mass and distribution. Sixty-five patients underwent a 2-hour oral glucose tolerance test for assessment of glucose tolerance using the American Diabetes Association diagnostic criteria [19]. Subjects were diagnosed with abnormal glucose tolerance if they met criteria for impaired fasting glucose, impaired glucose tolerance or diabetes mellitus based on the results of this test. All serum samples were analysed for sFRP2 levels. All patients gave written informed consent for inclusion in the study as approved by the appropriate research ethics committees. (National Research Ethics Service references 12/WM/1206 and 04/Q2707/278; University of Birmingham Biomaterials Ethics number 5389).

### Human serum assays

Human serum sFRP2 levels were measured using a commercially-available ELISA (USCNK Life Science Inc, B2 Scientific Limited, UK). Intrassay and interassay coefficients of variation (CV) for this assay were < 10% and < 12% respectively. Insulin was measured using a commercially-available colorimetric ELISA (Mercodia, Uppsala, Sweden) with an in-house CV of < 5%. Glucose, triglyceride and HbA1c were analysed on a standard automated platform (Roche Modular system, Roche, Lewes, UK). Fasting glucose and insulin levels were used in the homeostasis model assessment computer model (HOMA2-IR) to generate estimates of insulin sensitivity. HOMA%B is considered to be a measure of pancreatic beta-cell function and is expressed as a percentage [20].

### Dual energy X-ray absorptiometry (DXA) scanning

Body composition analysis was performed using Dual-energy x ray absorptiometry (DXA) with a total body scanner (9QDR 4500: Hologic, Bedford, MA). Coefficients of variation for multiple scans were 3%. Total fat (g) was calculated from the sum of arm, leg and trunk fat.

### Human Adipose Tissue (AT); Primary culture; Adipose Stromal Cell isolation

Paired primary human subcutaneous (SC) and omental (OM) adipocytes were isolated from adipose of healthy donors undergoing abdominal surgery for non-malignant, non-inflammatory conditions, from the University of Birmingham Human Biomaterials Resource Centre [12 males and 7 females; median body mass index (BMI) 29.0 kg/m^2^, range 20.8–37.9; age 62 years range 32–81] [21]. Briefly, adipose tissue samples were digested with 2mg/ml type 2 collagenase at 37°C for one hour followed by sterile filtration through layered gauze to remove solid components. After centrifugation, the stromovascular pellet containing preadipocytes was re-suspended in medium and plated in 12- or 24-well plates. Adipocytes and adipose-stromal cells (ASC) were separated by centrifugation at 100G for 5 minutes at room temperature. For experiments on preadipocytes, cells were grown to confluence and exposed to treatments in serum-free medium for 24 hours before biomaterial extraction. For testing of angiogenic gene mRNA expression these cells were treated with recombinant sFRP2 (rsFRP2) 400ng/ml for 48 hours (mouse rsFRP2 with 98% homology to human sFRP2, R&D Systems, Oxford, UK). This dose was chosen as it corresponds to a dose of 7.3nM for a 55kDa protein, which falls within the range used in previously-published work [16]. Subsequent experiments (see *de novo* lipogenesis and proliferation assay) were performed in human cell lines with concentrations that were more representative of the physiological circulating concentrations (see Results for circulating human concentrations). For experiments on mature adipocytes, preadipocytes were grown to 80% confluence before differentiation over 14 days in chemically-defined media into differentiated adipocytes.

### Adipose Tissue Conditioned Medium (ATCM)

To evaluate sFRP2 secretion from AT explants, 1g of paired SC and OM AT explants were incubated in serum free Dulbecco’s Modified Eagle’s Medium (DMEM) in air/5% CO_2_ at 37°C for 48 hours. AT was collected from 5 patients (2 male; BMI > 25 kg/m^2^). Proteins from ATCM were concentrated using Vivaspin columns (10,000 molecular weight cut-off value; Sartorius UK).

### Human preadipocyte cell line

The human Simpson-Golabi-Behmel syndrome (SGBS) preadipocyte cell line was obtained from Professor Martin Wabitsch at the University of Ulm. Cells were differentiated into adipocytes according to previously published protocols [22]. The SGBS cell line was derived from the stromal cell fraction of SC adipose tissue from a male infant with Simpson-Golabi-Behmel syndrome (SGBS), a rare X-linked congenital overgrowth syndrome. Proliferating SGBS cells were seeded in 12- or 24-well plates and grown to confluence in DMEM-F12 (Sigma Aldrich, Gillingham, UK) with 10% fetal calf serum (Life Technologies), additionally supplemented with 33μM biotin, 17μM pantothenate and penicillin-streptomycin 1% (growth media). In preadipocyte studies, cells were exposed to treatments, and biomaterial extracted, before differentiation. In adipocyte studies, SGBS cells were differentiated over 14 days in chemically defined medium. All cells were cultured in serum-medium for 24 hours before experiments.

### RNA extraction and Reverse Transcription

Total RNA was extracted from AT, adipocytes and ASC using TriReagent (Sigma-Aldrich, UK) according to the manufacturer’s protocol. RNA concentration was measured using a NanoDrop-1000 spectrophotometer (Agilent, UK) and integrity of RNA confirmed on 1% agarose gel. 500ng of total RNA was reverse transcribed using a High Capacity Reverse Transcription Kit (Applied Biosystems, UK) in 20 μl RT reaction as described in manufacturer’s protocol. cDNA was diluted to 10ng/ μl for PCR reactions.

### Conventional sFRP2 PCR

PCR reactions were carried out in 20μl volume using 40ng of cDNA, 10ng of each primer, at 60°C annealing temperature for 32 cycles. PCR products were separated on 1% agarose gel with 1:20,000 GelRed (Biotium, UK) and visualised using GeneSnap software (Syngene UK). All reagents were purchased from Promega (UK) unless specified otherwise.

Human sFRP2 primers sequence (345bp): *forward*: *CTGCCACCGCTTCACCGAGG*, *reverse*: *CCAGCCACCGAGGAAGCTCCA*.

Mouse sFRP2 primers sequence (320bp): *forward*: *ACGACAACGACATCATGGAA*, *reverse*: *GGAGATGCGCTTGAACTCTC*.

### Real time PCR (qPCR)

qPCR reactions were carried out in 20μl volume in 96-well plates with equal amount of cDNA (10ng) using ABI Prism 7500 Sequence Detection System (PE Applied Biosystems, UK). Housekeeping gene, 18S rRNA was used for relative gene expression. This enabled data to be expressed in relation to an internal reference to allow for differences in RT efficiency. Measurements were carried out at least three times for each sample. Primers and probe for all genes, including 18S rRNA as endogenous control, were purchased as the pre-made Expression Assays (Applied Biosystems, UK). According to the manufacturer’s guidelines, data were expressed as Ct values (the cycle number at which logarithmic PCR plots cross a calculated threshold line) and used to determine ΔCt values (ΔCt = Ct of the target gene minus Ct of the housekeeping gene). Relative gene expression data were calculated and presented as a fold increase over control. All statistics were performed on ΔCt values.

### Western blots on secreted sFRP2 protein from AT explants

Concentrated proteins from adipose tissue conditioned medium (ATCM) were separated on 10% acrylamide gel and immobilised to polyvinylidene membrane using semi-dry blot (Invitrogen, UK). They were immunoblotted overnight at 4°C with rabbit polyclonal sFRP2 antibody at 1:100 dilution (Milliprore, UK). Blots were incubated for 1 hour at room temperature with anti-rabbit secondary antibody at 1:20,000 dilution and specific bands visualised using Luminata Crescendo Western HRP Substrate (Millipore, UK). Western blot band intensity was measured using GeneSnap software (SynGene, Cambridge, UK).

### Mouse tissues

C57BL/6 mice aged 6–8 weeks (Jackson Laboratories, ME, USA) were housed in accordance with animal care protocols at the University of Birmingham, UK. Mice were maintained on a 12:12h light-dark schedule at 22°C, with up to 4 mice per cage. Mice were killed by cervical dislocation under terminal general anaesthesia using isofluorane prior to organ retrieval. Mouse gonadal fat (GF; equivalent of human OM tissue), pancreas and liver were collected in accordance with approved UK Home Office licences. Total RNA was extracted by homogenising tissues in TriReagent (Sigma-Aldrich, UK).

### *De novo* lipogenesis

Lipogenesis was measured by the uptake of 1-[^14^C] acetate into the lipid component of adipocytes [23]. After differentiation for 14 days, SGBS cells were washed and cultured in serum-free medium for 4 hours. Cells were then treated, with and without sFRP2 (50ng/ml and 100ng/ml), in serum-free medium for 18 hours. Hot (1-[^14^C]) and cold sodium acetate were then added to treatment wells for a further 6 hours. Cells were then washed three times and scraped with 250μl cold phosphate-buffered saline; cell lysate was transferred to glass thin layer chromatography capillary spotter tubes. Folch solvent (chloroform:methanol 2:1) 5ml was added to each tube to recover the lipid fraction; samples were mixed vigorously, followed by the addition of 1ml distilled water to each tube. Samples were separated into two phases after centrifugation at 12,000 for 5 minutes—aqueous (upper) and solvent (lower), with protein interface. The solvent fraction was recovered and transferred to scintillation tubes followed by evaporation to dryness overnight. Liquid scintillation cocktail (4ml, Fisher Scientific, Loughborough, UK) was added to each vial. Radioactivity retained in the cellular lipid was determined by scintillation counting and expressed as disintegrations per minute (DPM)/per well. Due to inter-assay variability, results were expressed as % change from controls per well.

### Proliferation study

SGBS preadipocytes were seeded into a 96-well plate. Following overnight culture cells were treated with 100ng/ml sFRP2 and compared to untreated controls. At Day 1, 3, 6, 8 and 10 cell proliferation was assessed using a nonradioactive cell proliferation assay (Promega, Madison WI) according to the manufacturer’s protocol. Luminescence was recorded at 490nm utilising a plate reader (Victor Wallac 3, Perkin Elmer). Results from controls were compared to sFRP2-treated cells using analysis of variance (ANOVA).

### Statistics

Statistical analysis was performed using IBM SPSS Statistics (IBM, Armonk, NY). Data were tested for normality using the Shapiro-Wilk test. Normally-distributed data were described using either mean and standard deviation (SD, clinical data), and mean and standard error of the mean (SEM, *in vitro* data), and compared using *t-*tests; data that were not normally distributed were described using median and range and compared using the Mann Whitney test. Correlation analysis was performed using Spearman’s rho test. Multiple linear regression was performed to confirm the relationship between clinical variables and sFRP2. ANOVA was used for analysis of the effect of sFRP2 on proliferation.

P values of 0.05 or less were considered statistically significant.

## Results

### Tissue-specific expression of sFRP2 in mouse and human tissues

**Fig 1:**
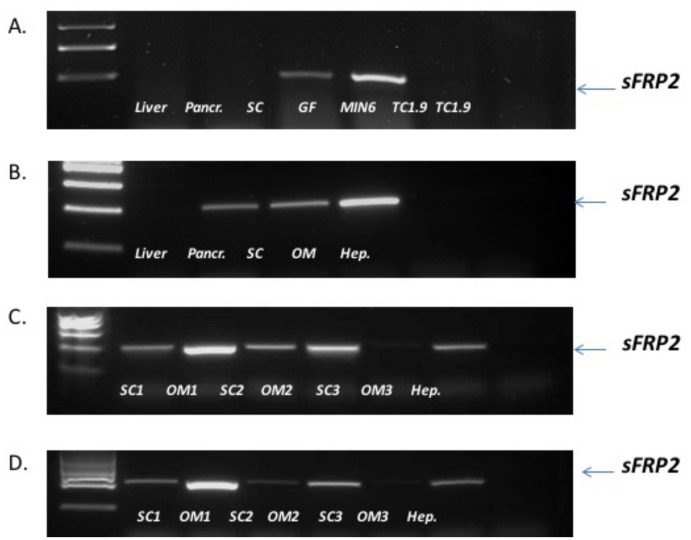
sFRP2 mRNA expression in mouse tissues: (A) liver, pancreas, SC AT, GF and beta and alpha pancreatic cell lines, (B) human tissues: liver, pancreas, SC and OM AT and isolated hepatocytes, (C) isolated adipocytes from human AT and (D) ASC cells isolated from human AT. PCR for 32 cycles. 18S PCR amplification was carried out for 20 cycles as control (data not shown).

RT-PCR on mouse tissues and cell lines revealed sFRP2 mRNA expression in mouse GF and SC fats but not in liver, pancreas and mouse pancreatic beta and alpha cell lines, MIN6 and TC1.9 respectively, Fig 1A. SFRP2 mRNA expression was detected in human pancreas, SC and OM AT but not in liver or isolated hepatocytes, Fig 1B. After digestion of human AT to isolate adipocytes and ASC, RT-PCR showed sFRP2 mRNA expression in human SC and OM adipocytes, Fig 1C, and ASC, Fig 1D. PCR for 32 cycles was indicative of higher expression in OM depots than SC AT.

### Depot-specific expression of sFRP2 in human adipose tissue

**Fig 2:**
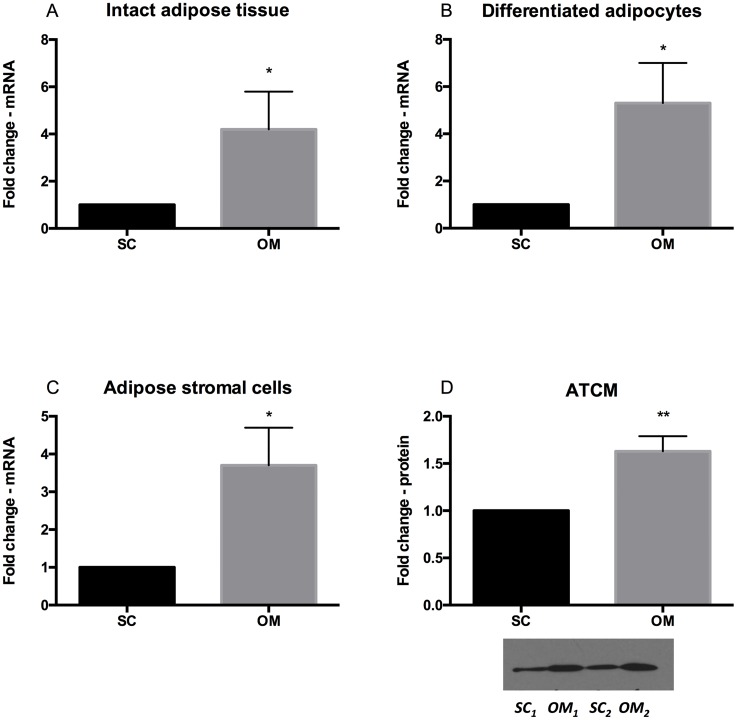
Relative sFRP2 mRNA expression by qPCR in (A) intact human SC and OM AT, (B) in isolated and differentiated human adipocytes and in (C) adipose stromal cells (ASCs). (D) Western immunoblot of sFRP2 shows band intensity (area) from adipose tissue conditioned media (ATCM) from 5 paired SC and OM explants after 48h incubation. An insert is a representative Western blot from 2 paired samples. ATCM was obtained by incubating the same amount of AT in 5ml media volume, n = 5 (D). *<0.05, **<0.01, ***<0.001.

The conventional PCR findings in human AT, adipocytes and ASC were confirmed by real time PCR. In intact human AT, sFRP2 mRNA expression was significantly higher in paired OM than SC depots (fold change 4.2 ± 1.6, p = 0.03, n = 4, Fig 2A). sFRP2 mRNA expression was also significantly higher in cells isolated and differentiated from OM compared to the SC depot (fold change, 5.3 ± 1.7, p = 0.02, n = 4, Fig 2B).

Similarly, in isolated ASC, OM ASC sFRP2 mRNA expression was significantly higher compared to corresponding SC expression (fold change 3.7 ± 1.0, p = 0.04, n = 4, Fig 2C). There was no significant difference in sFRP2 mRNA expression between adipocytes and ASC from the same AT depot, n = 8 (data not shown).

Western blot analysis for sFRP2 protein expression secreted into cell culture media revealed that OM AT explants secreted 63% more sFRP2 than SC explants during 48 hour culture (fold change 1.63±0.16, p<0.01, n = 5), Fig 2D.

### The effect of sFRP2 on proliferation, expression of angiogenesis-associated genes and on *de novo* lipogenesis

**Fig 3:**
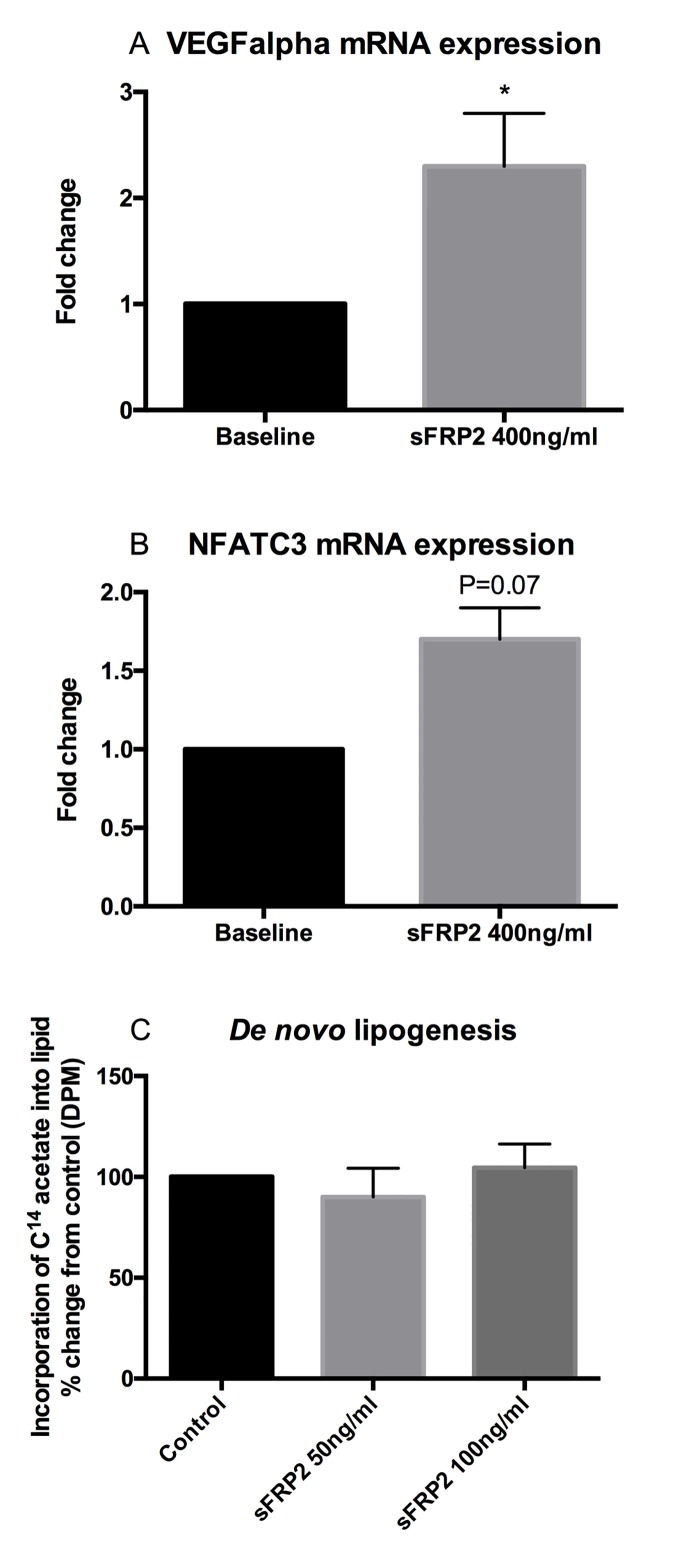
Angiogenic gene mRNA expression (A, VEGFalpha, B, NFATC3) in primary SC human preadipocytes treated with 400ng/ml sFRP2 for 48 hours. VEGFalpha mRNA expression was significantly increased by sFRP2 treatment. No effects were observed in OM preadipocytes (data not shown). Treatment with sFRP2 50ng/ml or 100ng/ml did not significantly increase *de novo* lipogenesis in differentiated SGBS cells (p = 0.5 and 0.7, respectively (C).

Treatment of SGBS cells with sFRP2 did not have any impact on proliferation compared to control SGBS cells (p = 0.6). Forty-eight hour treatment of primary SC human preadipocytes with sFRP2 led to increased expression of the angiogenic gene VEGFalpha (fold change 2.3 ± 0.5, p = 0.02, n = 5, Fig 3A). The observed increase in SC preadipocyte mRNA expression of NFATC3 after treatment with sFRP2 did not reach statistical significance (fold change 1.7 ± 0.2, p = 0.07, n = 5, Fig 3B). No effects on VEGFalpha or NFATC3 mRNA expression were observed in OM preadipocytes treated with sFRP2 (p = 0.22 and p = 0.37, respectively, n = 5, data not shown).

There was no effect of treatment with sFRP2 on *de novo* lipogenesis in differentiated SGBS cells [100% (control) v 90% ±24.8 (sFRP2 50ng/ml), p = 0.5; v 104% ±20.1 (sFRP2 100ng/ml), p = 0.7, Fig 3C].

### The relationship between sFRP2 and circulating insulin in human serum samples

**Fig 4:**
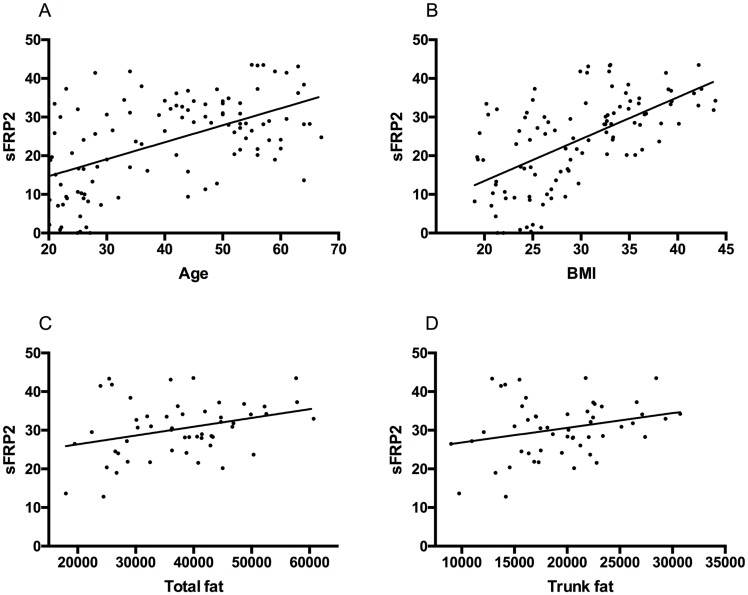
Correlation between (A) serum sFRP2 (ng/ml) with age (years) r = 0.29, p<0.0002; (B) BMI (kg/m^2^) r = 0.36, p < 0.0001; n = 106 (69 female); (C) total fat (g) r = 0.10, p = 0.02; and (D) trunk fat (g) r = 0.07, p > 0.05; n = 53.

In human serum samples, sFRP2 levels were positively correlated with increased age (r = 0.29, p<0.0002), BMI (r = 0.36, p < 0.0001) and triglycerides (r = 0.1, p = 0.01) but not HbA1c (p = 0.5). In the subset of subjects who underwent DXA scan measurement of fat depots, serum sFRP2 levels correlated with total fat (r = 0.10, p = 0.02); but not trunk fat (p > 0.05), Fig 4. Female subjects were found to have higher serum levels of sFRP2 than males (median 28.0, 0–43.5 ng/ml compared to 20.4, 0–42.0 ng/ml, p = 0.01).

**Fig 5:**
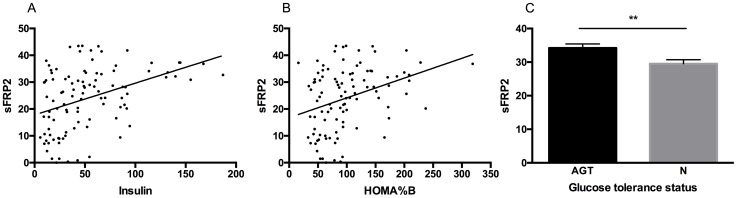
Correlation between (A) sFRP2 (ng/ml) and insulin (pmol/l), r = 0.16, p < 0.0001; (B) sFRP2 (ng/ml) and HOMA%B r = 0.12, p < 0.001; n = 106; (C) sFRP2 (ng/ml) and glucose tolerance status (abnormal vs normal glucose tolerance (AGT). Abnormal tolerance status comprises subjects with either pre-diabetes or diabetes on oral glucose tolerance test by ADA criteria), n = 65, mean+/-SEM.

Serum insulin (r = 0.16, p < 0.0001), HOMA%B (r = 0.12, p < 0.001) and HOMAIR (r = 0.15, p < 0.0001) correlated with sFRP2 levels in the human subjects, Fig 5A and 5B. In a multiple linear regression analysis of factors contributing to insulin secretion that included age and gender, only BMI (p <0.001) and sFRP2 (p <0.05) correlated significantly with circulating insulin concentration.

Because of the small numbers of subjects with diabetes (n = 8), these were combined with those diagnosed with pre-diabetes (n = 26) to create a category of abnormal glucose tolerance (AGT; n = 34). There was a significant difference between sFRP2 levels in those with normal (n = 31) (29.5; 16.9–43.1 ng/ml) compared to patients with abnormal glucose tolerance (34.2; 13.9–43.8 ng/ml, p < 0.01), Fig 5C.

## Discussion

We have characterised the adipose tissue depot-specific expression and secretion of sFRP2. We did not demonstrate an impact of recombinant sFRP2 on de novo lipogenesis nor on proliferation over a period of 10 days. Addition of recombinant sFRP2 to SC pre-adipocytes increased expression of VEGF which implies that in vivo sFRP2 may have a role in increasing blood supply to and expansion of adipose tissue volume. Unlike previous studies from a clinical setting [16], we measured circulating sFRP2 levels in serum from humans across a range of BMI and with normal and abnormal glucose tolerance and showed a positive correlation with serum insulin, triglyceride, HOMA%B and HOMAIR. Circulating sFRP2 was higher in female patients and in those with abnormal glucose tolerance and correlated with increased age and BMI. In a model of factors contributing to circulating insulin concentration, sFRP2 and BMI were the only variables which contributed significantly to the model.

In mouse tissues as well as in human, we identified expression of sFRP2 in adipose tissue, but not pancreas or liver. An earlier study identified sFRP2 by Northern blot in human pancreas using Clontech mRNA blots [15], but we were unable to detect its expression in mouse pancreas in agreement with a human study [24] detecting sFRP2 at very low level by RT-PCR. This suggests that any action of sFRP2 on the beta cell would be as an adipokine rather than any autocrine effect within the pancreas. Within adipose tissue, we hypothesised that sFRP2 might act as a paracrine / autocrine agent to stimulate de novo lipogenesis or proliferation of preadipocytes; this was not demonstrated. However, we observed an increase in VEGF gene expression suggesting a potential impact on angiogenesis. It is plausible therefore that total body fat might gradually increase over time in response to sFRP2-stimulated angiogenesis and increased blood flow to adipose tissue depots.

It was a limitation of the studies that the in vitro model was with cellular rather than tissue material, and thus an effect of increased angiogenesis within adipose tissue could not be evaluated. Another limitation of our study is that we did not assess Wnt pathway activation by sFRP2, to establish whether its action at the concentrations studied is that of an agonist or antagonist. However, the findings in our clinical study endorse the tight relationship between sFRP2, adipocyte biology and markers of insulin secretion and pancreatic beta cell function.

Human visceral obesity is strongly associated with cardiovascular risk [25, 26]. Our clinical study has shown a positive association between serum sFRP2, BMI and total fat; thus it is possible that sFRP2 from the visceral adipose depot might contribute to hyperinsulinaemia and cardiovascular risk. In animal models of heart failure, sFRP2 expression was increased, and blockade of sFRP2 using an antibody resulted in improved left ventricular ejection fraction [8]. The association of sFRP2 with cardiac fibrosis and with malignant neoplasms represents a potential common aetiology for the association of insulin resistance and diabetes with heart failure and with cancer.

Adipose tissue expression of sFRP2 has been associated with insulin resistance [16], and this study defined serum sFRP2 as an adipokine strongly associated with abnormal glucose tolerance and increased insulin secretion. Human obesity is often associated with insulin resistance, hyperinsulinaemia and beta cell dysfunction [27]; however the mechanisms are still not completely understood. Previous reports of sFRP2 stimulation of angiogenesis by the calcineurin pathway and inhibition of angiogenesis with antibody to sFRP2 [17] provide a clue to the link between adipose, insulin secretion and sFRP2; the calcineurin inhibitor tacrolimus inhibits sFRP2-induced VEGF expression and endothelial tube formation, reduces GLUT4 expression in adipocytes to render the adipocyte insulin resistant [28] and reduces beta cell mass [29]. It can be difficult to separate peripheral insulin resistance from compensatory hyperinsulinaemia in order to maintain euglycaemia—in this human study it should be noted that the overall cohort studied had normal insulin sensitivity with a median HOMAIR of 0.8 (0.1–4.7). It is possible that sFRP2-mediated adipose tissue expansion and insulin secretion is a potential compensatory mechanism in the setting of visceral adiposity and insulin resistance.

In conclusion, we have demonstrated differential adipose tissue specific expression of sFRP2 and have highlighted a potential role to drive adipose angiogenesis through enhanced VEGF expression that might explain our observed association between fat mass and circulating sFRP2 levels. Further studies are clearly warranted to determine, in vivo, the impact of sFRP2 manipulation on fat mass and adipose tissue biology, but it could represent a novel therapeutic target.

## Abbreviations

ANOVA: Analysis of variance
ASC: adipose-stromal cells
AT: adipose tissue
ATCM: adipose tissue conditioned medium
BMI: body mass index
C/EBPs: CCAAT enhancer binding proteins
CV: coefficient of variation
DXA: dual energy X-ray absorptiometry
ELISA: enzyme-linked immunosorbent assay
Frz: frizzled
GF: gonadal fat
HOMA: homeostatic model assessment, HOMA2 IR, for insulin sensitivity, HOMA%B, for insulin secretion
LRP5: low density lipoprotein receptor-related protein
mRNA: messenger ribonucleic acid
NFATC3: nuclear factor of activated T-cells 3
OM: omental
PPAR-gamma: peroxisome proliferator-activated receptor-gamma
SC: subcutaneous
sFRP: secreted frizzled related proteins
SGBS: Simpson-Golabi-Behmel syndrome
VEGF: vascular endothelial growth factor
Wnt: wingless/integrated
